# Correction to “Perioperative Outcomes of Robotic‐Assisted Versus Video‐Assisted Thoracoscopic Lobectomy: A Propensity Score Matched Analysis”

**DOI:** 10.1111/1759-7714.70398

**Published:** 2026-11-08

W. Zhiqiang and M. Shaohua , **“** Perioperative Outcomes of Robotic‐Assisted Versus Video‐Assisted Thoracoscopic Lobectomy: A Propensity Score Matched Analysis,**”** Thoracic Cancer 14(2023): 1921–1931, 10.1111/1759-7714.14938.PMC1034473637201914

In the originally published version of this article, several errors were identified.

In Figure 1, “Robotic‐assisted Lobectomy (*n* = 85)” and “Video‐assisted Lobectomy (*n* = 133)” should have appeared as “Robotic‐assisted Lobectomy (*n* = 88)” and “Video‐assisted Lobectomy (*n* = 120).” In addition, after propensity score matching, “Robotic‐assisted Lobectomy (*n* = 77)” and “Video‐assisted Lobectomy (*n* = 77)” should have appeared as “Robotic‐assisted Lobectomy (*n* = 71)” and “Video‐assisted Lobectomy (*n* = 71).” The correct figure is shown below.

**FIGURE 1:**
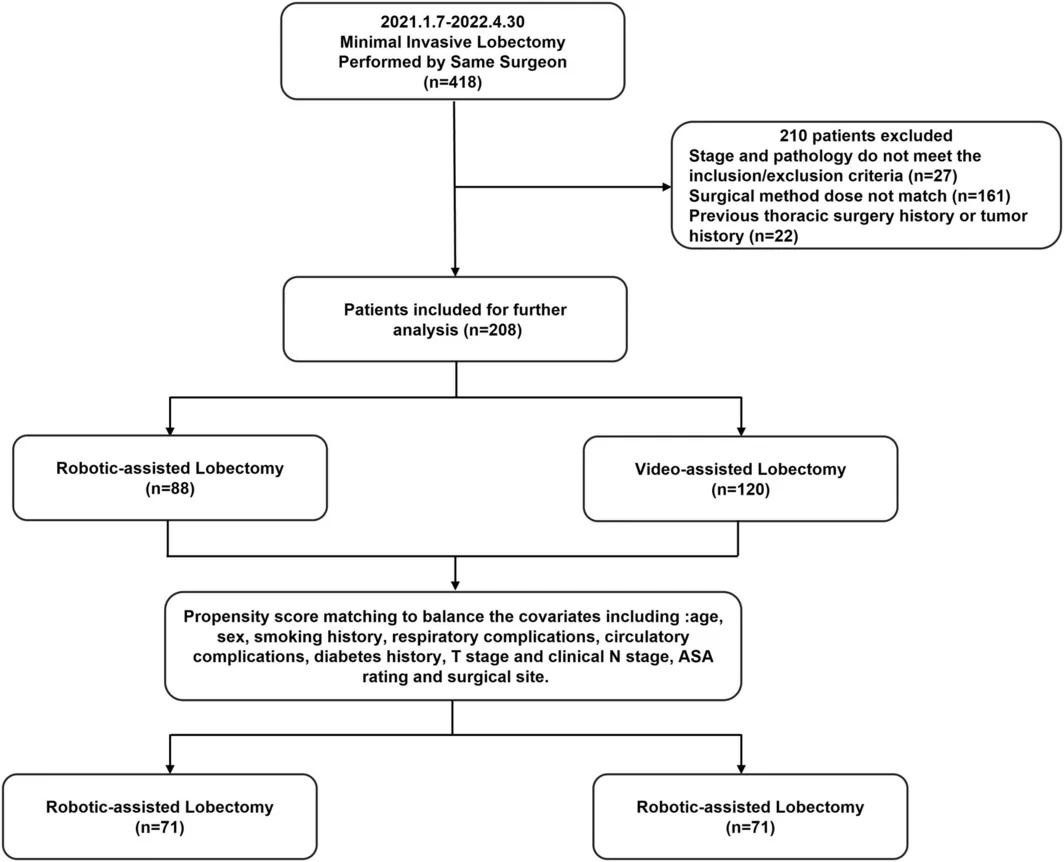
Flow chart of patients included in the study.

In the Patient selection section (page 1922, paragraph 4, line 2), “218 patients” should have appeared as “208 patients.”

In the Patient characteristics section (page 1923, paragraph 2, lines 4‐5), “133 patients” should have appeared as “120 patients,” and “85 patients” should have appeared as “88 patients.”

In Table 2, for the RATS group after PSM, the value for “No. of LNs, median(IQR)” was reported incorrectly as “10.0 (14.0–18.0)” and should have appeared as “10.0 (6.0–14.0).” In addition, the value for the “Yes” group under “Nodal upstaging, No. (%)” was reported incorrectly as “0 (0.0)” and should have appeared as “4 (5.6).”

We apologize for these errors.

